# Revisiting the Outcome of Displaced Two-Part Fractures of the Humeral Neck in Elderly Patients after Conservative Treatment

**DOI:** 10.5704/MOJ.1403.020

**Published:** 2014-03

**Authors:** L Bonifacio, P Syson, J Llanes

**Affiliations:** Philippine Orthopedic Center, Maria Clara St. corner Banawe Ave., Quezon City, Philippines; Philippine Orthopedic Center, Maria Clara St. corner Banawe Ave., Quezon City, Philippines; Philippine Orthopedic Center, Maria Clara St. corner Banawe Ave., Quezon City, Philippines

## Abstract

The aim of this study was to evaluate our experience with regard to the outcome of displaced two-part fractures of the humeral neck in elderly patients that were treated conservatively.

Between July 2008 and June 2010, 53 consecutive patients (42 females and 11 males; mean age = 74; range = 60-92) with an acute, displaced, two-part fracture of the humeral neck were treated conservatively using a sling and swathe for two weeks, followed by a standard rehabilitation protocol. The inclusion criteria were a displacement of the shaft >50% of its width and/or angulation of the shaft >45 degrees on standard radiographs. The exclusion criteria were patients younger than 60 years of age and those with cognitive or systemic impairment that would preclude the recommended physiotherapy. Patients were followed-up for one year, and were assessed at 3, 6, and 12 months using the Constant- Murley Score (CMS) and the Disabilities of the Arm, Shoulder, and Hand Questionnaire (DASH). Patients were divided into two groups, those below 70 years of age and those above 70-. Two-way repeated measures analysis of variance (ANOVA) was used to determine if there were significant differences between the results at 3, 6 and 12 months for both groups, and if the results were significantly different between the two groups.

Forty-eight out of 53 patients (91%) were able to complete the follow-up schedule, while five patients died. The mean CMS improved progressively at three (51.3), six (60.4), and 12 (61.3) months. The mean DASH also improved progressively at three (38.8), six (34.8), and 12 (32.6) months. For both groups, the CSS and DASH improved significantly from three to six months and from three to 12 months. However, the improvements were not significant from just six to 12 months. Between the two groups, the results at three, six, and 12 months were not significantly different from each other. On final follow-up, 42 out of 48 patients (88%) were satisfied with their outcome and reported that they would choose to undergo the same treatment if they had to do everything all over again.

Conservative management of displaced two-part fractures of the humeral neck in elderly patients is a safe, efficacious, and acceptable treatment.

## Introduction

Fractures of the proximal humerus account for 4-9% of all fractures, and 12.7% of these are two-part fractures of the surgical neck. These injuries have a unimodal distribution, usually occurring in patients with an average age of 72 years and with osteopenic bone^1^. It is estimated that 80% of these fractures are non-displaced, wherein conservative treatment is widely accepted due to good results^2^. However, for the remaining 20% that are displaced, the available literature mostly compares devices for operative treatment even though it remains unproven that surgery can give better results than conservative management^3^. Meanwhile, other papers either lump all kinds of proximal humerus fractures together or else focus on the rarer three-and/or-four-part fractures. The aim of this paper is to report our local experience with regard to the outcome of purely displaced two-part fractures of the humeral neck in elderly patients that were treated conservatively.

## Materials and Methods

### 

Between July 2008 and June 2010, all patients at least 60 years old seen at the emergency room with an acute, displaced, two-part fracture of the humeral neck were identified. They were all managed by a single orthopedic surgeon who is a co-author of this paper. The inclusion criteria, based on antero-posterior, lateral axillary, and scapular-Y radiographs, were a displacement of the shaft >50% of its width and/or angulation of the shaft >45 degrees in relation to the head fragment. The exclusion criteria were patients younger than 60 years of age and those with cognitive or systemic impairment that would preclude the recommended physiotherapy.

Upon seeing the patients at the emergency room, no reduction manoeuvre was attempted. Conservative treatment was instituted using a sling and swathe for two weeks of lenient immobilization. Unrestricted removal of the sling and swathe for hygiene or comfort was allowed as individually tolerated. Pain medications prescribed were arcoxia and tramadol as needed and dosed by weight. After 2 weeks, patients were begun on rehabilitation by a single physiotherapist thrice a week, with instructions to continue doing the taught procedures at home every day. The standard protocol started with pendulum exercises for two weeks. This was followed by gradual progressive activeassisted range of motion exercises for four weeks. Patients then came back for radiographic confirmation of union, defined as bridging callus formation on at least three cortices of the antero-posterior and lateral axillary views. Subsequently, active un-assisted range of motion exercises against gravity was initiated as tolerated for four weeks. Final radiographic confirmation of union was done at three months post-injury, after . After which, unrestricted use of the shoulder was allowed. Formal therapy was discontinued, but patients were still advised to continue doing the taught exercises at home every day.

Patients were followed-up for a total of one year post-injury, at three, six, and 12 months. All patients were assessed by a single orthopedic surgeon who is not a co-author of this paper. Two scoring systems were administered, the Constant-Murley Score (CMS)^4^ and the Disabilities of the Arm, Shoulder, and Hand Questionnaire (DASH)^5^. These two were chosen because they were commonly used in articles dealing with surgical treatment options for fractures of the proximal humerus. The CMS is specifically for shoulder function while the DASH is generally for upper extremity function.

The CMS consists of four domains: pain, activities of daily living (ADL), mobility, and strength. The items on pain and ADL are self-reported by the patient, while all other items are directly assessed by the examiner. The maximum score is 100, which means the best outcome. The single item on pain is scored from 0 (maximum pain) to 15 (no pain). The four items on ADL are scored from 0 (worst function) to 5 (best function). The four Items on mobility are scored from 0 (poorest function) to 10 (best function). The single item on strength is measured at 90 degrees lateral abduction by use of either an Isobex device or a defined spring balance technique: One point per 0.5 kg, with a maximum of 25 points.

In contrast to the CMS, the DASH consists of 30 items, six of which relate to symptoms (pain, weakness, stiffness, tingling or numbness) while 24 are about function (physical, social or role). All items are scored on a scale of 5, where higher scores mean better outcome: 1. no difficulty/symptoms 2. mild difficulty/symptoms 3. moderate difficulty/symptoms 4. severe difficulty/symptoms, and 5. extreme difficulty (unable to do)/symptoms. The maximum score is 150, which means the worst outcome.

## Results

A total of 53 consecutive elderly patients were collected, 42 female and 11 male, with ages ranging from 60-92 (mean = 74). Only 48 out of 53 patients (91%) were able to complete the one year follow-up schedule as five patients died.

On assessment, the mean CMS improved progressively at three (51.3), six(60.4), and 12 (61.3) months post-injury. The mean DASH also improved progressively at three (38.8), six (34.8), and 12 (32.6) months post-injury. If the five patients who died were included in an intention-to-treat analysis, the worst possible CSS would be 46.5, 54.7, and 55.5 respectively. On the other hand, the worst possible scores for DASH would be 44.6, 40.9, and 38.9 respectively. (See APPENDIX)

Patients were divided into two groups, those below 70 years old and those above 70 years old. Two-way repeated measures analysis of variance (ANOVA) was used to determine if there were significant differences between the results at three, six and 12 months for both groups, and if the results were significantly different between the two groups. For both groups, the CMS and DASH improved significantly from three to six months and from three to 12 months. However, the improvements were not significant from just six to 12 months. Between the two groups, the results at three, six, and 12 months were not significantly different from each other.

## Discussion

The scores for both CMS and DASH consistently improved on sequential assessments. The improvements post-injury were noted to be greater during the first six months compared with the second six months. This emphasizes the important role of early rehabilitation therapy to maximize this window for gaining improvement in function. Some studies have noted that there may be no difference in outcome between early mobilization (within one week postinjury) and late mobilization (at least three weeks of immobilization) at two years follow-up. However, there was still the finding of faster reduction of pain at three months in the early mobilization group^6^.

Furthermore, we believe that being aggressive to start early rehabilitation inspires patients to have a positive outlook on their prognosis by taking an active role in their healing process. It also counters speculative fears that they may have regarding causing inadvertently worse displacement or even non-union due to unintentional motion at the fracture site while doing common activities of daily living. The risk for non-union in these conservatively treated two-part humeral neck fractures was actually demonstrated by Court-Brown et al to be just 4.6%, regardless of the amount of initial displacement. They noted that advanced age (mean of 85 years in their study) may have a possible correlation with non-union, but their findings were not statistically significant1. In our study, there was no incidence of nonunion in the 48 out of 53 patients who were able to complete the final follow-up.

The available literature mostly compares devices for operative treatment of displaced two-part fractures of the humeral neck, even though it remains unproven that surgery can give better results than conservative management. Hauschild et al in 2013 conducted a prospective multi-centre cohort study comparing three different surgical methods (n = 133) and conservative management (n = 31). At final followup 12 months post-injury, they found no significant difference and concluded that the only benefit with operative treatment may be better range of motion and reduced pain in the first three months^3^.

The mean CS (74) that Hauschild et al obtained at 12 months may be slightly higher than the mean CMS (61.3), in the current study; on the other hand, the population was also older than theirs (mean age = 74 versus 62.9). In both studies’ CMS results for conservative management were even better or at least comparable to the results that Urda et al obtained in their 2012 study that also examined three different surgical methods for treating displaced two-part humeral neck fractures. In their 50 patients with a mean age of 70, although with the much longer mean follow-up period of 40 months, they obtained mean CMS scores of only 47.67 using K-wires, 72.72 using intramedullary nails, and 82.45 using locking plates^2^. Once again, evidence is lacking regarding whether surgery can give significantly better results than conservative management or otherwise.

In analyzing the results in the current study, the exact reason why some patients had lower CS and DASH scores than the others were unfortunately not determinable. Some possible hypotheses offered were: 1.) Presence of concomitant rotator cuff pathology, which could have even been present prior to the fracturecausing trauma and was quite common in the elderly. This could have been documented with magnetic resonance imaging, or at least ultrasonography. 2.) Possible non-compliance with the recommended daily home therapy, apart from the supervised sessions where as strict and consistent a regimen as possible were instituted. This may therefore correlate with improved final outcome for those patients who were more resolute and motivated to do well, because they would probably be able to maximize their improvement in time. 3.) Presence of osteoporosis, which could have impeded the resolution of pain expected with strengthening callus at the fracture site, thus slowing down the rehabilitation process. This could have been documented with bone mineral density studies. 4.) Severity of initial fracture displacement and angulation, as well as final resultant malunion. These measurements were difficult to obtain accurately on standard radiographs, but the possible correlation of increasing severity with worsening outcome would have been advantageous to document.

**Table I:** 

Months Post-Injury	3	6	12
Mean CS	51.3 + 16.1	60.4 + 18.4	61.3 + 21.6
Worst possible CS	46.5	54.7	55.5
* Higher scores mean better outcome			
Mean DASH	38.8 + 21.8	34.8 + 23.5	32.6 + 25.2
Months Post-Injury	44.6	40.9	38.9
* Higher scores mean worse outcome			

**Fig. 1: Antero-posterior radiograph of a case with satisfactory result.**
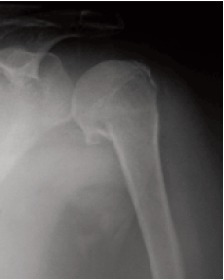


**Fig. 2: Axillary radiograph of a case with satisfactory result.**
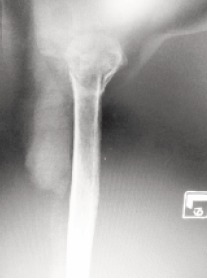


**Fig. 3: Antero-posterior radiograph of a case with unsatisfactory result.**
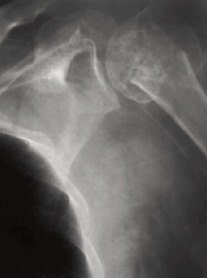


**Fig. 4: Axillary radiograph of a case with unsatisfactory result.**
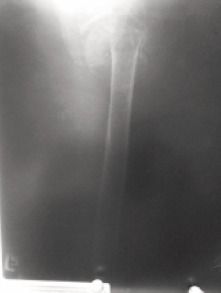


## Conclusion

Conservative management of displaced two-part fractures of the humeral neck in elderly patients is a safe, efficacious, and acceptable mode of treatment. On final follow-up at 12 months post-injury, 42 out of 48 patients (88%) were satisfied with their outcome and reported that they would choose to undergo the same treatment if they had to do everything all over again. More comparative studies between conservative and operative management may be needed before justifying the added morbidity and expense associated with surgical intervention. Further studies could also address the limitations encountered in this study, especially the lack of ancillary procedures that could have helped point out the reasons for some patients having poorer outcome than others at final follow-up.

## References

[1] Court-Brown CM, Garg A, McQueen MM (2001). The translated two-part fracture of the proximal humerus: Epidemiology and
Outcome in the Older Patient.. J Bone Joint Surg (Br).

[2] Urda A, Gonzalez A, Colino A, Lopiz Y (2012). Management of displaced surgical neck fractures of the humerus: health related
quality of life, functional and radiographic results. Injury, Int. J. Care Injured.

[3] Hauschild O, Konrad G, Audige L, de Boer P, Lambery SM, Hertel R (2013). Operative versus non-operative treatment for twopart
surgical neck fractures of the proximal humerus. Arch Orthop Trauma Surg.

[4] Constant and Murley (1987). A Clinical Method of Functional Assessment of the Shoulder. Clin Orthop Relat Res.

[5] (2013). Institute for Work and Health (IWH). The DASH Outcome Measure: Disabilities of the Arm, Shoulder, and Hand.. http://www.dash.iwh.on.ca.

[6] Lefevre-Colau, Babinet A, Fayad F, Fermanian J (2007). Immediate Motion Compared with Conventional Immobilisation for the
Impacted Nonoperatively Treated Proximal Humeral Fracture.. J Bone Joint Surg Am.

[7] Keser S, Bolukbasi S, Bayar A, Kanatli U, Meray J, Ozdemir H (2004). Proximal humeral fractures with minimal displacement
treated conservatively. International Orthopaedics (SICOT).

[8] Zyto, Karol (1998). Non-operative treatment of cornminuted fractures of the proximal humerus in elderly patients. Injury.

[9] Marie-Jeanne TFD, Peeters Vrancken, Kastelein Gerard Willem, Breslau1 Paul John (2001). A Prospective Study of the Functional
Outcome after Conservative Treatment.. Eur J Trauma.

[10] Karataglis D, Stavridis SI, Petsatodis G, Papadopoulos P, Christodoulou A (2011). New trends in fixation of proximal humeral fractures:
A review. Injury, Int. J. Care Injured.

